# Relationship between type of skin lesions and nailfold capillaroscopy pattern in mixed connective tissue disease

**DOI:** 10.1007/s10067-021-05717-4

**Published:** 2021-08-09

**Authors:** Anna Felis-Giemza, Sylwia Ornowska, Ewa Haładyj, Zenobia Czuszyńska, Marzena Olesińska

**Affiliations:** 1 Department of Connective Tissue Diseases, National Institute of Geriatrics, Rheumatology and Rehabilitation, Warsaw, Poland; 2 Department of Internal Medicine, Connective Tissue Diseases and Geriatrics, Medical University of Gdansk, Gdansk, Poland

**Keywords:** Connective tissue disease, Microscopy, Microvascular damage, Scleroderma, Skin

## Abstract

**Introduction:**

Mixed connective tissue disease (MCTD) is a rare disease with clinical picture consisted of multiple organ manifestations, including skin changes resembling systemic lupus erythematosus (SLE), systemic sclerosis (SSc), or dermatomyositis (DM). On the background of these manifestations are microvascular changes — alteration of endothelial function and impairment of endothelial progenitor cell. Nailfold capillaroscopy (NFC) is a simple, non-invasive technique for investigating microvascular involvement in rheumatic diseases.

**Objectives:**

To describe the relationship between type of skin lesions and NFC pattern in MCTD patients.

**Methods:**

We analyzed the clinical picture and NFC patterns in 79 patients with MCTD. The NFC changes were classified into Normal, “Early,” “Active,” and “Late” scleroderma-like patterns (SD-like pattern) based on Cutolo classification.

In all patients, subjective and physical examinations were carried out, specifically the occurrence of skin lesions in the course of MCTD was assessed (systemic sclerosis-like (Ssc-like), systemic lupus erythematosus-like (SLE-like), dermatomysitis-like (DM-like)).

**Results:**

Skin changes were present in 64 (81%) patients, involving 43 (54%) SLE-like, 48 (61%) SSc-like, and 4 (5.1%) DM-like. NFC changes were observed in a total of 55 (69.6 %) patients with predominance of the “Early” pattern — 41 (51.9 %) patients. According to skin change phenotypes, NFC changes were observed in 31 (72%) patients with SLE-like and in 32 (66.7%) patients with SSc-like skin phenotypes. The “early” pattern predominated in both group.

**Conclusions:**

We did not find any correlation between NFC pattern and the type skin changes.

**Table:** 

**Key Points** *• The study did not show a correlation between the presence and absence of skin lesions and NFC pattern.* *• Scleroderma-like patterns were found in over 60% of patients with mixed connective tissue disease.* *• The “early” pattern is dominant regardless of the occurrence or absence of skin lesions in patients with MCTD.* *• Skin lesions, regardless of their type (SLE or SSc), do not correlate with type of lesion found in the NFC examination.*

## Introduction

Mixed connective tissue disease (MCTD) is a rare systemic autoimmune disease with an overlapping feature of at least two connective tissue diseases (CTDs), including systemic lupus erythematosus (SLE), systemic sclerosis (SSc), polymyositis (PM), dermatomyositis (DM), and rheumatoid arthritis (RA), with the presence of antibodies against U1-ribonucleoprotein (RNP), especially at high titers [1–4]. In the literature, the most frequently observed manifestation is RP, found in 75–90% of patients. It may precede other clinical symptoms of the disease for several months or even years [5]. In the majority of patients with MCTD, skin symptoms typical for at least one of the diseases constituting its clinical picture are present. [1].

The cause of the disease is unknown. Damage to small vessels plays an important role in the pathogenesis of the disease. The endothelial progenitors cells become disabled and its functions are impaired [6–8].

Nailfold capillaroscopy (NFC) is an easy, safe, and non-invasive technique for qualitative and quantitative assessment of microcirculation of the distal capillary row [9]. NFC is widely used in the diagnosis of rheumatic diseases, as well as in evaluating and differentiating primary and secondary RP [10]. NFC is particularly useful in the diagnosis of SSc. The microvascular changes seen in NFC in SSc patients are referred to as scleroderma pattern (SD-pattern) and have three distinctly different types, ranging from “Early” through “Active” to “Late,” according to the Cutolo classification [11]. Numerous studies have shown that in patients with SSc, local changes in microcirculation and their type reflect the severity of organ lesions and disease and that there is a correlation between disease progression and capillaroscopic image progression over time [12]. In other connective tissue diseases such as mixed connective tissue disease, overlap syndromes, dermatomyositis, and polymyositis, scleroderma-like pattern (SD-like pattern) was observed [15–17]. Early detection of organ involvement enables earlier initiation of treatment and improved outcomes in MCTD patients.

The aim of this study is to determine whether the occurrence of skin lesions in patients with MCTD coexists with the occurrence of lesions in the NFC examination and whether a specific type of skin lesion, characteristic of SSc or SLE, is associated with a specific capillaroscopic image of the nail shaft vessels.

## Materials and methods

The study was conducted with a group of patients hospitalized in the years 2007–2011 in two rheumatological centers in Poland: the Connective Tissue Disease Department of Institute of Geriatrics, Rheumatology and Rehabilitation in Warsaw and Department of Internal Medicine, Connective Tissue Diseases and Geriatrics, Medical University of Gdansk in Gdansk. Prior to the study, the Bioethical Committee of the Institute of Rheumatology approved its conduct. All human research procedures followed were in accordance with the Helsinki Declaration of 1975, as revised in 2013. Before the start of the study, each patient was presented with the aim and methods of the study and written consent to participate in the study was obtained. The study involved 79 patients with MCTD. The diagnosis of the disease was established on the basis of Kasukawa’s diagnostic criteria [18]. All of patients met the diagnostic criteria. Based on the medical history and available documentation, data on the onset and course of the disease were collected.

Three types of onset of the disease (time between the appearance of the first symptoms of MCTD and its full clinical picture) were distinguished: acute, where this period was less than 2 weeks; subacute, where it lasted from 2 weeks to 2 months; chronic, where it exceeded 2 months[19]. Three types of disease manifestation were determined: single phase, when after initial exacerbation, there was a period of remission; multiphase, with repeated periods of exacerbation and remission; chronic, when following initial symptoms of the disease, there was no remission. In all patients, interview and physical examinations were carried out; specifically, the occurrence of skin lesions in the course of MCTD was assessed. Next, MCTD patients were divided into 3 groups according to the skin lesions typical for particular disease units constituting the clinical picture of MCTD: SLE (butterfly-shaped rash, hair loss, UV sensivity, and livedo reticularis), SSc (sclerodactyly, telangiectasias), and DM (heliotrope erythema, mechanic’s hands, Gottron’s sign, swollen eyelids, swollen nose).

In all 79 patients with MCTD, a capillaroscopic examination of finger nail shafts was performed. Each patient was allowed to adapt to environmental conditions for about 15–20 min before the examination. The first series of vessel loops of nail shafts of all fingers from II to V excluding the thumb were evaluated. Immersion oil was used to improve visibility [14, 18]. The study at the Centre in Gdansk was carried out with the MOTIC 168, the study in the center in Warsaw using the SZ2-ILST (Olympus) at a final magnification of ×200. The qualitative assessing the microcirculation of the distal capillary row of all patients was assessed by the same expert. The capillaroscopy findings were classified into non-scleroderma pattern (without abnormalities, called normal in text) or scleroderma-like pattern and then describe as “Early,” “Active,” or “Late” based on Cutolo classification [20–22]. Scleroderma-like pattern is characterized with giant capillaries (diameter > 50 μm), hemorrhages, derangement, avascular areas, and neoangiogenesis [17]. Patients with normal capillary image and with scleroderma-like pattern were included in the study.

Normal distribution of continuous variables was verified using the Kolmogorov-Smirnov test. As most continuous variables were not distributed normally, Mann-Whitney *U*-test was used for intergroup comparisons. The distributions of discrete values were compared with Pearson’s chi-square test or Fisher’s exact test. All statistical calculations were performed using Statistica 10 (StatSoft, Tulsa, OK, USA), taking *p* ≤ 0.05 as statistical significance level.

## Results

### Study group

The study group consisted of 79 patients with MCTD, including 66 (83.5%) women and 13 (16.5%) men — the number of women (W) was almost 5 times higher than men (M) (5.03:1). All of patients fulfilled the Kasukawa’s diagnostic criteria. The median age of patients during the study was 44 years (18–67). The most frequent symptoms at disease were skin lesions, articular and muscular symptoms, and hematological disorders (Table 1). Skin symptoms were found in 64 (81%) patients. Changes typical for SLE and SSc occurred with similar frequency in 43 (54%) and 48 (61%) patients, respectively, and typical for DM only in 4 (5.1%) patients. Fourteen (18%) patients had skin lesions typical of lupus, with no coexistence of other types. Skin symptoms typical for systemic scleroderma, without the presence of other types of lesions, were found in 21 (27%) patients in the study group. In 25 (32%) cases, SLE-like skin lesions were accompanied by skin lesions typical for SSc, in 2 (2.5%) cases — lesions typical for DM. In 2 patients (2.5%), there was a co-occurrence of skin lesions typical for SLE, SSc, and DM. In many cases, skin lesions typical for more than one disease were found, while in 1/5 of patients, no such lesions were found at all. More often, the coexistence of ≥ 2 types of skin lesions (in different combinations) than one type was observed.

**Table 1:** Clinical symptoms in MCTD patients *n* = 79

Skin lesions	*n* (%)	Artricular and muscular	*n* (%)
SLE-like skin lesion	43 (54.4)	Arthritis	21 (26.6)
DM-like skin lesion	4 (5.0)	Arthralgia	35 (44.3)
SSc-like skin lesion	48 (60.8)	Erosions	3 (3.8)
“Puffy fingers”	37 (46.8)	Deformation	12 (15.2)
Raynaud’s phenomenon	75 (94.9)	Myalgia	19 (24.1)
		Weakness of the proximal muscles	12 (15.2)
Respiratory system		Neurological system	
Pleural involvement	3 (3.8)	Stroke	3 (3.8)
↓TLCO	11 (13.9)	Epilepsy	1 (1.3)
Pulmonary arteria hypertension	12 (15.2)	Depression	5 (6.3)
Interstitial lung disease	2 (2.5)	Involvement of the cranial nerves	12 (15.2)
		Involvement of peripheral nerves	11 (13.9)
Cardiovascular system		Hematological disorders	
Arterial hypertension	23 (29.1)	Leukopenia	43 (54.4)
Pericardial involvement	2 (2.5)	↑ γ-globulin	37 (46.8)
Conduction abnormalities	12 (15.2)	Lymphopenia	33 (41.8)
Nephropathy		↓ Hemoglobin level	13 (16.5)
Proteinuria > 0.5g/d	2 (2.5)	Thrombocytopenia	6 (7.6)
Proteinuria + erythrocyturia	2 (2.5)		
Other			
Sjӧgren’s syndrome	24 (30.4)	Esophagus involvement	20 (25.3)

Abbreviation: *MCTD*, mixed connective tissue disease

Patients with skin lesions compared to patients without them did not differ in terms of demographic data. The median age of patients was about 40 years, with the incidence rate falling in the third decade of life. The duration of the disease was 7–9 years and its course was most often chronic progressive (61% in patients with skin lesions and 60% in patients without skin lesions). Seventy-five patients (94.9%) had RP in the whole study group. In patients with skin lesions (SLE, SSc, DM) was presented in 60 (93.8%), while among the group lacking skin lesions, all patients had it.

In the whole group of MCTD patients, SD-like pattern was observed in 55 (69.6%) patients. In 41 (51.9%) patients, changes characteristic for “Early” pattern according to Cutolo were found. “Active” and “Late” patterns were less frequent, in 8 (10.1%) and 6 (7.6%) patients, respectively.

### Group of MCTD patients with skin lesions

Sixty-four (81%) MCTD patients had skin lesions. The capillaroscopic damage occurred in 44 (68.8%) patients in this group, the “Early” pattern predominated. The results are presented in Table 2.

**Table 2:** NFC characteristics in MCTD patients with or without skin lesions

NFC changes	Patients with skin changes (*n* = 64; 81%)	Patients without skin changes (*n* = 15; 19%)	*P*
No changes in NFC	20 (31.3%)	4 (26.7%)	0.73
Any NFC abnormalities	44 (68.8%)	11 (73.3%)	0.27
Early pattern	31 (48.4%)	10 (66.7%)	0.20
Active pattern	7 (11%)	1 (7%)	0.62
Late pattern	6 (9%)	0 (0%)	0.22

Abbreviation: *NFC*, nailfold capillaroscopy; *MCTD*, mixed connective tissue disease

### Group of MCTD patients without skin lesions

There were no skin lesions in 15 (19%) patients with MCTD. Changes in capillaroscopic examination were found in 73.3% (11%) of patients in this group. Similarly, as in the group of patients with skin lesions, “Early” pattern dominated. The results are presented in Table 2.

Based on the study, no significant correlation was found between the presence or absence of skin lesions in patients with MCTD and the presence and type of lesions following a capillaroscopic examination (Table 2).

### Analysis of MCTD patients with SLE-like skin lesions (*n* = 43)

The analysis of the group of patients with SLE-like skin lesions compared to the group of patients without such lesions is presented in Table 3. RP was present in almost all patients in this group (40; 93%). Based on the analysis, statistically significant differences in the duration and course of the disease were observed between patients with SLE-like skin lesions and patients without the presence of these skin lesions.

**Table 3:** Comparison of demographic and clinical data in patients with MCTD with (SLE+) and without (SLE−) skin lesions typical of SLE

Parameter	SLE+, *n* = 43	SLE−, *n* = 36	*P*
Gender			
F (%)	88.4	77.8	0.169
M (%)	11.6	22.2	
Age (years)			
Median (min.–max.)	45 (19–67)	41.5 (18–62)	0.386
Average ± SD	44.5 ± 13	41.6 ± 13.2	
Age of onset (year)			
Median (min.–max.)	32 (6–66)	34.5 (12–56)	0.434
Average ± SD	31.4 ± 14.1	33.6 ± 13.7	
Disease duration (months)			
Median (min.–max.)	132 (12–420)	84 (10–264)	0.024
Average ± SD	142.2 ± 106.9	88.3 ± 63.5	
Disease course: *n* (%)			
Single phase	5 (12)	2 (6)	0.296
Multiphase	18 (42)	6 (17)	0.014
			0.004
Type onset of disease n (%)			
Acute	13 (30)	7 (19)	0.522
Subacute	25 (58)	25 (70)	
Chronic	5 (12)	4 (11)	

Abbreviation: *MCTD*, mixed conenctive tissue disease; *SLE*, systemic lupus erythematosus

Among 43 patients with SLE-like skin lesions, capillaroscopic lesions were observed in a majority of patients with the prevalence of “Early” pattern. Based on the study, no significant correlation was found between the occurrence of SLE-like skin lesions and the presence or type of lesions on capillaroscopic examination in MCTD patients. The results are presented in Table 5.

### Analysis of a group of MCTD patients with SSc-like skin lesions (*n* = 48)

The analysis of the group of patients with SSc-like skin lesions compared to the group of patients without such lesions is presented in Table 4. Similarly, to the group of patients with lesions typical for SLE, the occurrence of RP was found in the majority of patients, at 93.8%. Among 48 patients with SSc-like skin lesions, changes in capillaroscopic examination were observed in 32 patients (66.6%). The “Early” pattern dominated. The results are presented in Table 5. Based on the results of the study, it was determined that the compared groups of patients did not differ significantly in terms of gender, age, age of onset, as well as its duration and course.

**Table 4:** Comparison of demographic and clinical data in patients with (SSc+) and without (SSc−) skin lesions typical of scleroderma

Parameter	SSc+, *n* = 48	SSc−, *n* = 31	*P*
Gender			
F (%)	87.5	77.4	0.352
M (%)	12.5	22.6	
Age (years)			
Median (min.–max.)	46 (18–67)	41 (18–66)	0.252
Average ± SD	44.6 (13.2)	41.1 (12.7)	
Age of onset (year)			
Median (min.–max.)	33 (6–66)	33 (8–51)	0.968
Average ± SD	32.5 (15.1)	32.3 (12.0)	
Disease duration (months)			
Median (min.–max.)	116 (11–420)	84 (10–300)	0.092
Average ± SD	134.1 ± 102.6	92.2 ± 70.7	
Disease course: *n* (%)			
Single phase	3 (7)	4 (13)	0.353
Multiphase	13 (27)	11 (35)	
Chronic progressive	32 (66)	16 (52)	
Type onset of disease *n* (%)			
Acute	8 (17)	12 (39)	0.036
Subacute	32 (66)	18 (58)	0.480
Chronic	8 (17)	1 (3)	0.081

Abbreviation: *SSc*, systemic sclerosis

**Table 5:** Characteristics of the capillaroscopic image depending on the type of dominant skin lesions in MCTD patients

NFC changes	SLE+	*P*	SSc+	*P*
NFC without abnormalities	12 (28%)	0.60	16 (33.3%)	0.48
NFC abnormalities	31 (72%)	0.46	32 (66.7%)	0.27
Early pattern	21 (48.8%)	0.55	21 (44%)	0.07
Active pattern	5 (11.6%)	0.63	6 (12.5%)	0.38
Late pattern	5 (11.6%)	0.13	5 (10.4%)	0.24

Abbreviation: *MCTD*, mixed connective tissue disease; *NFC*, nailfold capillaroscopy; *SLE*, systemic lupus erythematosus; *SSc*, systemic sclerosis

There was also no significant correlation between the occurrence of SSc-like skin lesions and the presence or type of lesions in capillaroscopic examination in patients with MCTD.

## Discussion

Skin symptoms are easily detectable by physical examination or a thoroughly documented medical history. The type of skin lesion and the degree of their severity may make the diagnosis of the disease easier. Skin lesions observed in patients with SSc are very characteristic of this disease, enabling the diagnosis of SSc [23]. Similarly, skin symptoms are considered important during the development of MCTD classification criteria [24]. The overall picture of skin lesions in patients with MCTD compared to those with SSc is more varied. Within the study group, most (81%) of the patients with MCTD had skin lesions, which is consistent with observations of other authors [2, 25, 26]. In a study by Szodoray et al. on 201 Hungarian patients, 96% of them had skin lesions in the form of UV sensitivity, a butterfly-shaped rash, telangiectasia, and hyper/hypopigmentation [27]. In the present study, skin symptoms were classified as typical for SLE, SSc, and DM. The literature describes individual skin lesions found in patients with MCTD, but so far, their frequency has not been described in order to specify changes typical for SLE and SSc. The most common changes typical of SLE in patients with MCTD include a butterfly-shaped rash, which occurs, depending on disease activity, 30–53% of the time [28]. In SSc, however, sclerodactylia is a common symptom found in 55.2% of patients with MCTD [27]. Skin symptoms typical for DM, such as Gottron’s papules or heliotrope erythema, are rarely found in patients with MCTD, as demonstrated by the study group [29]. When determining the study group divisions that is according to the type of skin lesion, qualifying a given patient to more than one group if there were skin symptoms characteristic for more than one disease was permitted. This constitutes a detriment of the study, resulting mainly not only from statistical requirements but also from the heterogeneity of clinical symptoms in MCTD.

In over 80% of SSc patients, abnormalities in capillaroscopic examination were observed [20, 30–32]. The changes in NFC have been so characteristic of SSc that the presence of SD-patterns has been included in the new EULAR/ACR classification criteria for this disease since 2013 [23]. The SD-pattern in NFC is diagnosed early in patients with SSc and may precede other symptoms or even diagnosis of SSc for many years. It is also clear that in patients with SSc, changes in microcirculation and their type reflect the severity of the disease and intensity of organ abnormalities. Numerous studies have shown a correlation between the disease progression and changes in capillaroscopic image over time. SSc patients with a “Late” pattern in NFC have a higher risk of severity and progression of skin lesions as well as organ complications [12, 13, 33]. The literature concerning mainly patients with SSc suggests a relationship between the occurrence of changes in nail shaft microcirculation (e.g., reduced capillary density and neoangiogenesis) and the development of lung disease or pulmonary arterial hypertension in these patients [34–37]. In patients with SLE, no typical changes in capillaroscopic examination have been identified so far, and an SD-like pattern in NFC is rarely found. “Non-specific” lesions are found most often, and in about 30% of patients, mild lesions in the form of tortuosity or elongated loops may occur. Additionally, in SLE patients hemorrhages may occur more often than compared to healthy patients. However, the occurrence of SD-like pattern in patients with SLE was associated with a higher incidence rate of pulmonary arterial hypertension than compared to patients with SLE with a normal capillaroscopic image. Cutolo et al. cite studies describing the correlation between capillaroscopic changes and disease activity [38, 39]. In contrast to patients with SSc, in patients with MCTD, changes in capillaroscopic examination are less frequent. The literature suggests the occurrence of SD-like pattern in NFC in almost 60–70% of patients with MCTD among the remaining ones, “non-specific” changes or a normal image are found [31, 40, 41]. Moreover, there is little data on the relation between the capillaroscopic image and organ activity or damage in MCTD patients. There are single reports indicating the relation between the presence of capillaroscopic changes characteristic of SSc and the incidence of lung disease or muscle involvement in patients with MCTD [31, 41, 42]. In our observations, microvascular damage was found in over 60% of patients with MCTD, similarly as it is described in the literature [31]. In the studied group of patients with MCTD, skin lesions, regardless of their type, do not correlate with the presence or type of lesion found in the NFC study. In the evaluated group, SD-like pattern occurs both in patients with SSc-like and SLE-like skin lesions. In both groups of patients, the “Early” pattern predominated. The “Early” pattern is dominant regardless of the occurrence or absence of skin lesions in patients with MCTD and regardless of the type of skin involvement (SLE/SSc). Almost all patients in the study group used glucocorticosteroids, and some of them additionally used other immunosuppressive medicines such as methotrexate, azathioprine, hydroxychlorochine/chlorochine, and endoxan. It remains unclear whether immunosuppressive therapy affects the microvascular changes observed in NFC. The literature suggests that the treatment may affect microcirculational changes [31].

In some patients with MCTD, we observe similar changes in capillaroscopic examination in the form of scleroderma-like pattern as in patients with Ssc. In our study, as in the works of other authors, this is about 60% of patients. Therefore, we wonder whether, similarly to SSc patients, capillaroscopic changes in MCTD patients may correlate with disease progression and organ involvement. There are isolated studies showing an association of organ involvement with capillaroscopic findings in MCTD. The presence of SSc-like skin lesions in MCTD patients does not suggest that the course of the disease will be similar to SSc patients. It remains unclear whether SSc-like skin lesions in patients with MCTD occurring in early stages of the disease will also dominate in the later stages and whether, as in patients with SSc, will be related to the manifestation and progression of internal organ involvement (e.g., lung involvement and pulmonary arterial hypertension). The study we have presented did not include the involvement of other organs. However, as there are few reports on capillaroscopic imaging in MCTD patients, our study may be useful in furthering and gaining knowledge about this rare disease and NFC in MCTD patients. There are few reports on this topic in the literature, the disease is rare, and the groups studied are usually small. More research is needed on this topic. If similar correlations to SSc are found, capillaroscopy could be useful in diagnosis and perhaps predicting disease progression and organ involvement in MCTD patients. Since capillaroscopy is an easy and inexpensive examination with no adverse effects, it can be performed in patients with MCTD without any harm, both in those with and without skin lesions. Lack of skin lesions in patients with MCTD does not exclude the occurrence of changes in NFC. It would enable us to analyze more groups of patients and their capillaroscopic images, providing additional information about the disease. Therefore, we believe this study is worth performing in all patients with MCTD. NFC, as a non-invasive and easily accessible tool, could be used to assess disease activity or organ involvement in MCTD patients following longer observation. Early diagnosis of organ involvement enables earlier initiation of treatment and improved outcomes in MCTD patients.

Currently, there are no validated methods for assessing the activity or damage index of MCTD. All assessments are only estimates. The majority of publications concerning MCTD use scales designed for SLEs (SLEDAI for activity assessment, SLICC/ACR DI for damage assessment) [43]. It is worth mentioning that this study included a small sample size, which beckons the need for further, larger prospective studies. In addition, we realize that the limitation of the study is the performance of a qualitative analysis.

The study did not show a correlation between the presence and absence of skin lesions, regardless of their type (SSc or SLE), nor the SD-like patterns in NFC in the group of MCTD patients. Even more frequent changes in NFC occurred in patients without any skin lesions, but no statistical significance was demonstrated. Currently, there are no data on abnormalities in capillaroscopic examination during the disease and their relation to the activity or progression of the disease in patients with MCTD. Therefore, observations should be repeated after 5 years on patients with MCTD in order to assess the changes over time in the capillaroscopic examination in comparison with the clinical evaluation of MCTD.
